# Diet and Genotype of an Aquatic Invertebrate Affect the Composition of Free-Living Microbial Communities

**DOI:** 10.3389/fmicb.2020.00380

**Published:** 2020-03-17

**Authors:** Emilie Macke, Martijn Callens, Francois Massol, Isabel Vanoverberghe, Luc De Meester, Ellen Decaestecker

**Affiliations:** ^1^Aquatic Biology, IRF Life Sciences, KU Leuven, Kortrijk, Belgium; ^2^Centre d’Ecologie Fonctionnelle et Evolutive, UMR CNRS 5175, Montpellier, France; ^3^CNRS, Lille–Sciences et Technologies, UMR 8198 Evo-Eco-Paleo, SPICI Group, Villeneuve-d’Ascq, France; ^4^University of Lille, CNRS, INSERM, CHU Lille, Institut Pasteur de Lille, U1019–UMR 8204–CIIL–Center for Infection and Immunity of Lille, Lille, France; ^5^Aquatic Ecology, Evolution and Conservation, KU Leuven, Leuven, Belgium

**Keywords:** microbiome, *Daphnia*, bacterioplankton, community structure, ecosystem level effects

## Abstract

In spite of the growing interest in the role of the gut microbiome (GM) in host physiology and health, the mechanisms governing its assembly and its effects on the environment are poorly understood. In this article, we show that the host genotype and the GM of *Daphnia* influence the community structure of the surrounding bacterioplankton (BPK). When *Daphnia* genotypes were placed in an identical environment, both the GM and BPK showed a genotype and diet-dependent taxonomic composition. Overall, the GM strongly differed from the BPK in taxonomic composition and was characterized by a lower α-diversity, suggesting a selective rejecting of bacteria from the regional species pool. In a microbiome transplant experiment, the assembly of both the GM and BPK was strongly affected by the host genotype and the inoculum to which germ-free *Daphnia* were exposed. The combination of these results suggests a strong interaction between the host genotype, its GM and free-living microbial communities. Currently, it is generally assumed that an animal’s diet has a strong effect on the animal’s GM, but only a negligible (if any) effect on the surrounding environment. However, our results indicate that the diet/microbiome inocula have a small effect on the gut community and a large effect on the community in the surrounding environment. This structuring genotype × microbiome × environment effect is an essential prerequisite that could indicate that microbiomes play an important role in eco-evolutionary processes.

## Introduction

The composition of the gut microbiome (GM) is characterized by a strong host-specific dominance of particular taxa, but with substantial variability over time within one host individual (Dabrowska and Witkiewicz, 2016; Adair and Douglas, 2017; Zhang et al., 2017). A key challenge is to decipher the factors shaping the host’s microbiome community and the relative contribution of different deterministic and stochastic processes to variation in its community structure (Alberdi et al., 2016; Adair and Douglas, 2017). So far, most studies on GM assembly have focused on within-host processes (Macke et al., 2017b; De Meester et al., 2019). Host-mediated control over the GM composition has been shown to occur in several taxa, most often due to antimicrobial defenses mounted by the host (Tasiemski et al., 2015) or developmentally regulated anatomical barriers to colonization (Ohbayashi et al., 2015; Lanan et al., 2016; Norman and Koskella, 2017). Behavioral traits such as selective feeding or sociality also contribute to shaping the GM by modifying the pool of potential colonizing microbes (Grossart et al., 2010; Koch and Schmid-Hempel, 2011; Mushegian et al., 2019). These deterministic processes partly depend on the host’s genetic background, suggesting an association between host genotype and GM composition (Macke et al., 2017a; Mushegian et al., 2019). On the other hand, microbe-microbe interactions, including competition and syntrophic interactions, have also been shown to affect the persistence of microbes within the gut (Adair and Douglas, 2017; Foster et al., 2017; Macke et al., 2017a). These interactions are strongly mediated by the host’s diet, which determines the available resources in the gut and, accordingly, what microbial species and types of metabolism can become dominant (Carmody et al., 2015; Foster et al., 2017).

The GM does not exist in isolation, but can contribute to a wider metacommunity maintained by the transmission between individual hosts and dispersal between host-associated and free-living microbial communities (Eckert et al., 2016; Murall et al., 2017). Dispersal of microbes is likely to be key in maintaining microbiome diversity and reducing variation in microbiome composition between individual hosts (Alberdi et al., 2016; Adair and Douglas, 2017; Macke et al., 2017b). Hence, processes involved in structuring the GM might often involve multiple spatial scales (Penczykowski et al., 2016; Norman and Koskella, 2017; Laine et al., 2019). In this way, the GM could be regarded as a local community colonized from a regional species pool, i.e., the microbial pool present in the environment (Shapira, 2016; Foster et al., 2017). The composition of this regional pool depends on environmental conditions, but might also be directly affected by hosts and their microbiomes (Wong et al., 2015; Callens et al., 2018). The interaction between hosts, their microbiomes and free-living communities is expected to play a crucial role in microbiome assembly. This metacommunity perspective on microbiome assembly might be particularly relevant for aquatic organisms and bacterioplankton (BPK), which are in close and continuous contact and are therefore expected to have strong reciprocal effects. If these interactions prove to be host genotype-dependent to a certain extent, then they may also be an important missing link in eco-evolutionary theory (Macke et al., 2017b; De Meester et al., 2019).

Combining metabarcoding with a GM transplant approach, we investigated the interplay between host genotype, GM, and BPK in the freshwater crustacean *Daphnia magna*. In a first experiment, we examined the effects of *Daphnia* genotype and diet on both the GM and BPK composition. Nine *Daphnia* genotypes were exposed to an environmental microbial source and were submitted to either a green algal or a cyanobacterial diet for approximately 58 generations. GM and BPK compositions were subsequently determined through next-generation sequencing and were found to be affected by host genotype and diet. In a second experiment, we examined the relative contribution of the microbial source *versus Daphnia* genotype-dependent sorting processes to GM and BPK assembly by performing a GM transplant. Germ-free *Daphnia* of two genotypes were exposed to different GM inocula. Two weeks after the transplant, GM and BPK compositions were assessed through next-generation sequencing, revealing a combined effect of microbial source and host genotype on the structuring of both communities.

## MATERIALS AND METHODS

### *Daphnia magna* Genotypes

Nine *Daphnia* genotypes (G1–G9) were used in Experiment 1. A subset of these genotypes (G2, G6, and G9) was used in Experiment 2. G1, G4, and G9 were originally isolated from an 8.7 ha shallow man-made pond located in Oud-Heverlee, Belgium. Clonal lineages were established from resting eggs sampled in three sediment core sections (18–21 cm, 11–14 cm, and top 3 cm depth, respectively), corresponding to three time periods (1970–1972, 1976–1979, and 1988, respectively). G3 was hatched from the top 3 cm sediment of a small, fishless and mesotrophic pond (350 m^2^) located near Knokke, Belgium (51°20′05.62″ N, 03°20′53.63″ E). G5–G8 were hatched from the top 3 cm sediment of a 3.7 ha eutrophic pond containing fish and located in Heverlee, Belgium (50°51′47.82″ N, 04°43′05.16″ E). G2 was isolated from Bysjön lake in Sweden. All genotypes were maintained in the laboratory under standardized stock conditions for several years prior to the experiment, in aged tap water, and for the two last years in re-constituted freshwater (ADaM medium) (Klüttgen et al., 1994), at a temperature of 19 ± 1°C and under a 16:8 h light:dark cycle, in 500 ml glass jars containing 20–30 individuals. The *Daphnia* were fed twice a week (0.8 mg C/L) with the green alga *Scenedesmus obliquus*; the medium was partly refreshed a few times a year and regularly replenished to compensate for evaporation. As all genotypes were maintained in the laboratory for several years prior to the experiment, we consider it unlikely that the microbiota of the stock cultures were dominated by bacteria from the pond of origin. However, we cannot exclude this possibility, given that recent research has indicated that some bacterial species are shared between adults and resting eggs (Mushegian et al., 2018).

### Sterile Cultures of Green Algae and Cyanobacteria

The unicellular green alga *S. obliquus* (hereafter called *Scenedesmus* or abbreviated as “S”; strain CCAP 276/3A, provided by the Culture Collection of Algae and Protozoa, United Kingdom) and the unicellular cyanobacteria *Microcystis aeruginosa* (hereafter called *Microcystis* or abbreviated as “M”; strain PCC 7806, provided by the Pasteur Culture Collection, Institut Pasteur, Paris, France) were used to feed the *Daphnia*. *Scenedesmus* and *Microcystis* differ in their nutritional value and digestibility (von Elert et al., 2003). The *Microcystis* strain used in the present study produces toxins and bioactive compounds such as microcystins (Rohrlack et al., 2001). The *Scenedesmus* and *Microcystis* were grown in WC medium (Guillard and Lorenzen, 1972) and modified WC medium (WC medium without Tris), respectively, under sterile conditions at 20 ± 2°C and a light:dark cycle of 16:8 h in 2 L glass bottles with constant stirring and aeration. Filters (0.22 μm) were placed at the input and the output of the aeration system to avoid bacterial contamination. Algae were harvested weekly in their early stationary phase. Axenity was checked on LB medium agar plates. The ash-free dry weight of the cultures was determined according to Moheimani et al. (2013).

### Experiment 1. Effects of *Daphnia* Genotype and Diet on Bacterioplankton and Gut Microbiome Composition

Previous research has already examined the GM composition of four genotypes (G1, G3, G7, and G8, see Macke et al., 2017a, Experiment 1) but in our experiment we extended the GM characterization from four to nine genotypes (G1–G9) and followed the population for a much longer time. We also included BPK analyses.

For each of the nine *Daphnia* genotypes, three maternal lines were cultured in ADaM medium (Klüttgen et al., 1994) in 2 L experimental glass jars, under standardized conditions (19 ± 1°C; 16:8 h light:dark cycle). They were fed daily with saturating amounts of *Scenedesmus*. Medium was refreshed once a week. When a sufficient number of individuals was reached, 120 juveniles were sampled from each maternal line and divided into two 2 L experimental jars (each containing 60 individuals). The first jar was fed a *Scenedesmus* diet, while the second was fed a *Microcystis* diet. In total, there were 54 populations (9 genotypes × 2 diets × 3 replicates). Algae were provided every other day with a final carbon concentration of approximately 1.5 mg C.L^–1^ according to (Macke et al., 2017a). Medium was refreshed every other week. Water from a pond situated in the ECOLAB on the campus (Kortrijk, Belgium, 50°48′30.3″ N, 3°17′38.0″ E) was added to the ADaM medium (15% of the final volume) every 2 weeks in order to provide a large diversity of bacteria and optimal growth conditions for the *Daphnia*, as detected in (Callens et al., 2016, 2018).

After 1.5 years (*circa* 58 generations) of exposure to the two types of diet, the composition and diversity of the BPK and GM community were assessed through next-generation sequencing of 16S rRNA. To obtain GM samples, 20 adult *Daphnia* were collected from each population and placed in a sterile (i.e., autoclaved) ADaM medium for 24 h to reduce the number of contaminating food particles within the gut (Callens et al., 2016). The *Daphnia* guts were subsequently extracted using sterilized dissecting needles under a stereomicroscope and jointly placed in 1.5 ml Eppendorf tubes containing 10 μL of deionized sterile water. For BPK characterization, 100 ml of medium was sampled from each population and filtered with a 0.22 μm syringe filter. The filter was subsequently placed in a 1.5 ml Eppendorf tube. GM and BPK samples were immediately placed at −20°C until further processing.

### Experiment 2. Effects of Microbial Source and Host Genotype on Bacterioplankton and Gut Microbiome Assembly in a Microbiome Transplant Experiment

Previous research has already examined the GM composition of the recipient genotypes (Macke et al., 2017a, Experiment 3). Here, we extended the analyses to the surrounding BPK. *Daphnia* genotypes G2 and G9 were used as GM donors in the transplant experiment. For each genotype, six replicated populations were grown in 2 L jars and divided into two groups submitted to different experimental conditions in order to maximize variation in GM composition. In the first condition, the diet was composed of *Microcystis*, and filtered water from a pond situated on the university campus (ECOLAB, Kortrijk, Belgium, 50°48′30.3″ N, 3°17′38.0″ E) was added to the ADaM medium (5% of the final volume) every 2 to 3 weeks, to provide *Daphnia* with a diverse pool of microbes. In the second condition, the diet was composed of *Scenedesmus* and water from another pond on campus was added to provide *Daphnia* with another set of environmental bacteria. In this experiment, we made diverse inocula to examine whether *Daphnia* genotypes would consistently select for particular strains from these different inocula. However, by doing so the diet effect might be confounded by a pond effect, which is in line with our focus on microbiome effect in Experiment 2/shifting our focus from the diet effect to the microbiome inoculum effect in Experiment 2. Algae were provided every other day as in Macke et al. (2017a). After 8 weeks of exposure to the two conditions, 60 adult *Daphnia* individuals (i.e., 20 per replicate) were collected for each genotype and their GM was extracted. This way, we obtained four different microbial inocula, each one containing the GM of 60 *Daphnia* and representing the factorial combinations of donor genotypes (G2 and G9) and diets (*Microcystis* and *Scenedesmus*). These inocula are referred to as G2M, G2S, G9M, and G9S.

Twelve maternal lines were then created for each recipient genotype (G2 and G6) and grown separately in 500 ml jars for several generations on a diet of saturating amounts of *Scenedesmus*, under the conditions described above. Due to the loss of the G9 clone during the preparation of the transplant experiment, no full reciprocal transplant was possible, therefore G9 was replaced by G6. From each maternal line, germ-free individuals were obtained as described in Macke et al. (2017a). For each maternal line of each recipient clone, 40 germ-free juveniles were jointly placed in a 50 ml centrifuge tube containing 45 ml of sterile ADaM. The 12 tubes were divided into four groups, each one inoculated with either a “G2M,” “G2S,” “G9M,” or “G9S” microbial inoculum (on average one crushed gut for four germ-free recipient *Daphnia*) and 1 mg C.L^–1^ of *Scenedesmus*. *Daphnia* were left in these conditions for 2 days to ensure that they ingested enough bacteria and were subsequently transferred to a 500 ml jar containing fresh sterile ADaM medium. They were then submitted to an 80% *Microcystis* – 20% *Scenedesmus* diet with a final carbon concentration of 1 mg C.L^–1^. These algae were provided every day. Pond water was not added in this transplant experiment. In total, there were 24 recipient populations (2 recipient genotypes × 4 microbiome inocula × 3 replicates) with 40 *Daphnia* juveniles in each population. Two weeks after the transplant, a sample of medium (100 ml) and ten *Daphnia* guts were collected from each recipient population for BPK and GM characterization, using the protocols described in (Macke et al., 2017a).

### Determination of Bacterioplankton and Gut Microbiome Community Composition and Diversity

DNA was extracted using a PowerSoil DNA Isolation Kit (MO BIO Laboratories) and dissolved in 20 μL MilliQ water. For Experiment 1, we obtained 108 DNA samples (9 genotypes × 2 diets × 3 replicates × 2 community types). For Experiment 2, we obtained 48 samples (2 recipient genotypes × 4 microbiome inocula × 3 replicates × 2 community types). DNA yield was determined using a Qubit dsDNA HS Assay Kit (Invitrogen) on 3 μL of the sample. Four samples from Experiment 1 (GM of G2–M, BPK of G5–M, and BPK of G5–S) and one sample from Experiment 2 (Recipient G2 – Inoculum G2S) did not contain sufficient DNA and were discarded. Because of the initially low bacterial DNA concentration in some samples, a nested PCR was applied to increase specificity and amplicon yield (Callens et al., 2016). The full length 16S rRNA gene was amplified with primers 27F and 1492R on 10 ng of template (94°C – 30 s; 50°C – 45 s; 68°C – 90 s; 30 cycles) by using a high-fidelity Pfx Polymerase (Life Technologies). PCR products were subsequently purified with the QIAquick PCR Purification Kit (Qiagen). To obtain dual-index amplicons of the V4 region, a second amplification was performed on 5 μL PCR product using primers 515F and 806R for 30 cycles (94°C – 30 s; 55°C – 30 s; 68°C – 60 s). Both primers contained an Illumina adapter and an 8-nt barcode at the 5′-end. For each sample, PCRs were performed in triplicate, pooled and gel purified using the QIAquick Gel Extraction Kit (Qiagen). An equimolar library was prepared by normalizing amplicon concentrations with a SequalPrep Normalization Plate Kit (Applied Biosystems) and subsequent pooling. Amplicons were sequenced using a v2 PE500 kit with custom primers (Kozich et al., 2013) on the Illumina MiSeq platform (KU Leuven Genomics Core) producing 2 × 250-nt paired-end reads.

Sequence reads were processed using R 3.3.2 (R Core Team, 2018) as previously reported by Callahan et al. (2016b). Sequences were trimmed (the first 10 nucleotides and all nucleotides from position 190 onward were removed) and filtered (maximum two expected errors per read) on paired ends jointly. Sequence variants were inferred using the high-resolution DADA2 method (Callahan et al., 2016a) and chimeras were removed. Taxonomy was assigned with a naive Bayesian classifier using the RDP v14 training set. Amplicon sequence variants (ASVs, hereafter called OTUs) which had no taxonomic assignment at phylum level or were assigned as “Chloroplast” or “Cyanobacteria” were removed from the dataset. For Experiment 1, the final dataset contained 2,887,669 reads, on average 27,766 reads per sample (minimum = 4,133 reads, maximum = 63,594 reads). For Experiment 2, the final dataset contained 1,500,800 reads, on average 29,427 reads per sample (min. = 5,804 reads, max. = 78,154 reads).

Statistical analyses and plots were performed using R 3.3.2 (R Core Team, 2018). To investigate differences in community composition among samples (β-diversity), Bray–Curtis dissimilarity indices were calculated and plotted using Principal Coordinates Analysis (PCoA) with the phyloseq package (McMurdie and Holmes, 2013). For Experiment 1, the effects of diet, genotype, community type (i.e., BPK and GM) and their interactions on β-diversity were assessed through a PERMANOVA, using the Adonis function of the vegan package (tested using 999 permutations of rows). For Experiment 2, we also used a PERMANOVA to examine how microbiome inoculum, recipient genotype, community type, and their interactions might affect community composition. For both experiments, analyses were carried out both for GMs and BPKs jointly and for each of them separately. Samples and taxa were clustered hierarchically based on Bray–Curtis dissimilarity indices, using the UPGMA (unweighted pair group method with arithmetic mean) agglomeration method, with the fastcluster package. A heat map of OTUs dominance in the different samples was obtained using the gplots package. To identify how bacterial classes differed among community types and diets, OTUs were grouped at class level, and differential abundance analyses were performed with the Bioconductor package DESeq2 (Love et al., 2014). Venn diagrams illustrating overlap of OTUs among community types and diets were produced using Venny 2.1.0 online freeware (Oliveros, 2007).

For both experiments, we investigated the correlation between variation in BPK composition on the one hand and variation in GM composition on the other hand by performing a Mantel test to compare the Bray–Curtis dissimilarity matrices of the two community types (permutations = 999; statistics based on Pearson’s correlation). Ordinations (i.e., PCoA) of BPK and GM were compared through a Procrustes analysis of the first two axes, and significance was assessed though permutation tests (999 permutations) with the protest function of the vegan package.

For α-diversity, OTU richness (total number of OTUs) was calculated using the vegan package. In Experiment 1, the effects of diet, genotype, community type, and their interactions on OTU richness were assessed through generalized linear models (GLM) with a Poisson distribution. *p*-values were adjusted for multiple comparisons through the control of the false discovery rate (FDR). When interactions with community type have proven to be significant, analyses were also performed separately for BPK and GM. Pairwise comparisons among genotypes and diets were performed by contrasting least-squares means. For Experiment 2, we also used GLM to test for the effects of microbiome inoculum, recipient genotype, community type, and their interactions on OTU richness. Similar analyses, which are presented in the Supplementary Material, were performed on the Shannon diversity index.

## Results and Discussion

**FIGURE 1:**
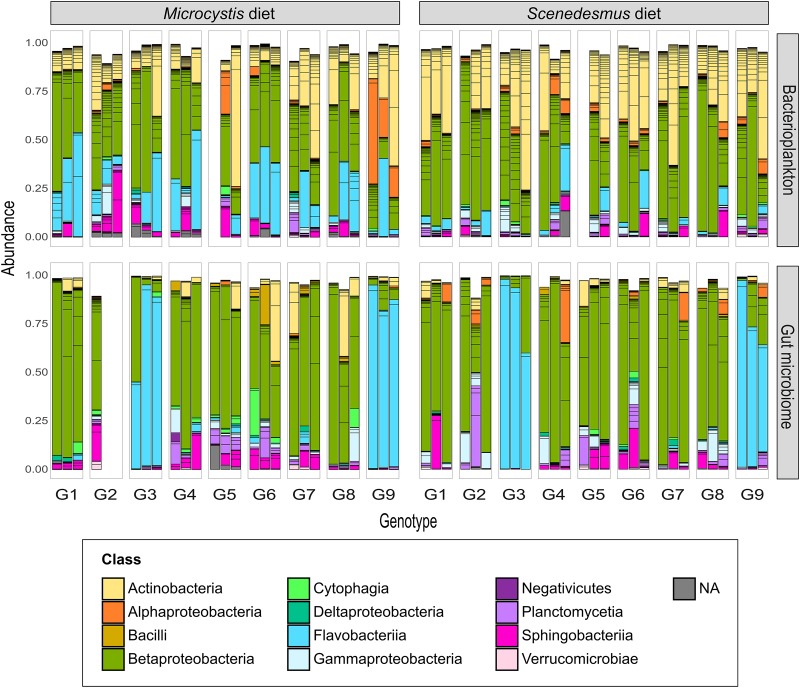
BPK and GM composition across *Daphnia* genotypes and diets (Experiment 1). Nine *Daphnia* genotypes (G1–G9) were exposed to either a *Microcystis* or a *Scenedesmus* diet. After 58 generations of exposure, both *Daphnia* GM and BPK were characterized through next generation sequencing (*n* = 3 replicated populations per genotype × diet combination). Bars indicate the relative abundance of OTUs. Colors indicate bacterial classes. OTUs with an occurrence lower than 1% are not represented.

**FIGURE 2:**
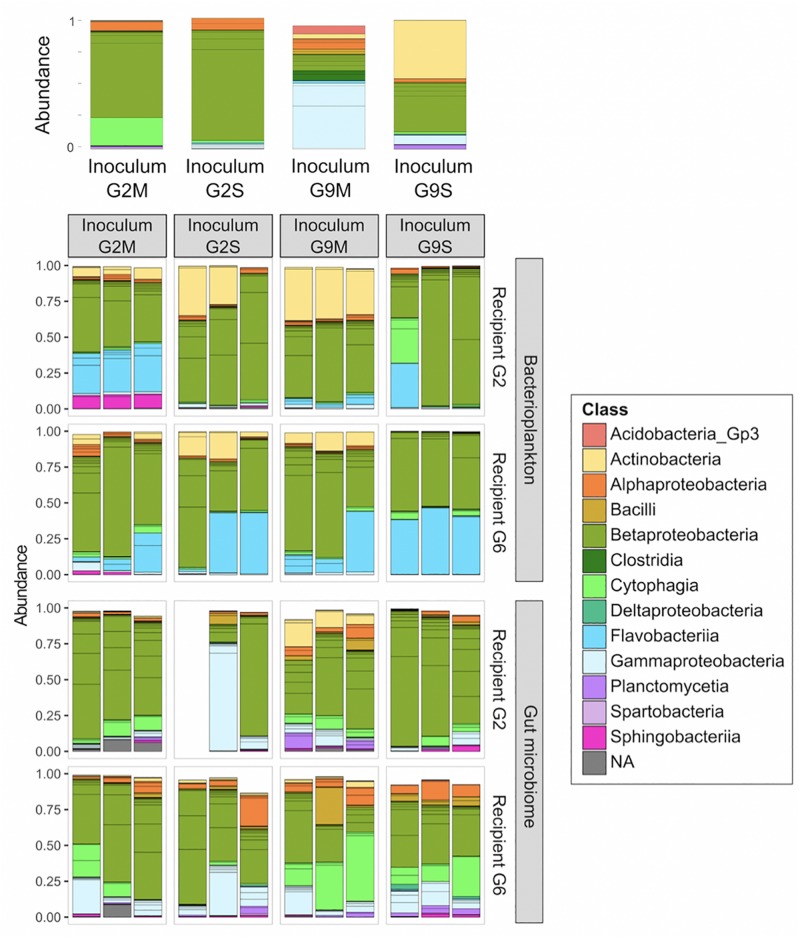
BPK and GM composition in recipient *Daphnia* genotypes following a GM transplant (Experiment 2). Two germ-free recipient *Daphnia* genotypes (G2 and G6) were inoculated with four types of microbial inocula (G2M, G2S, G9M, and G9S). Two weeks after the transplant, both *Daphnia* GM and BPK were characterized through next generation sequencing (*n* = 3 replicated populations per recipient genotype × inoculum combination). Bars indicate the relative abundance of OTUs. Colors indicate bacterial classes. OTUs with an occurrence lower than 1% are not represented. Upper panel is the GM composition of the inocula.

**FIGURE 3:**
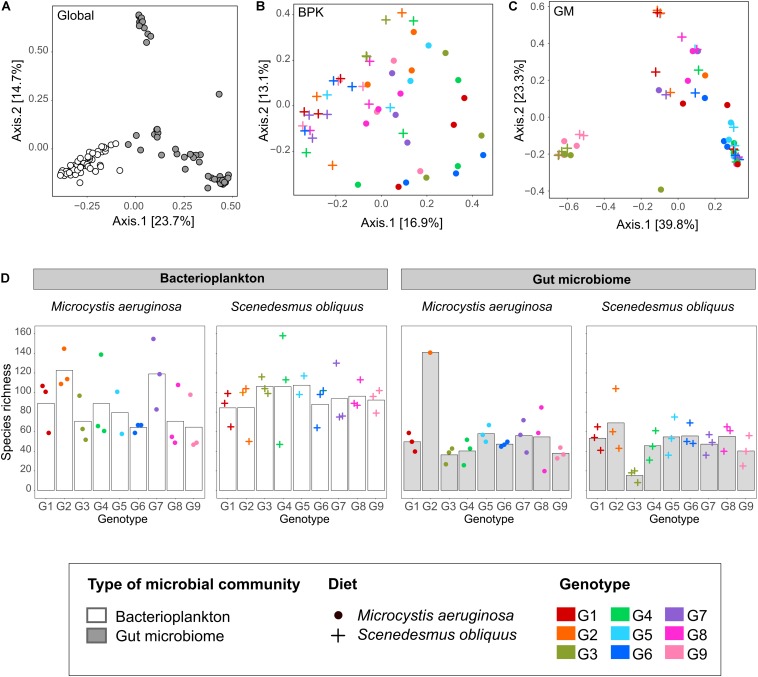
*Daphnia* genotype and diet effects on GM and BPK community structure (Experiment 1). **(A–C)** PCoA using Bray–Curtis dissimilarity indices considering all samples simultaneously **(A)**, BPK samples only **(B)**, and GM samples only **(C)**. **(D)** α-diversity (OTU richness) in GM and BPK when *Daphnia* genotypes (G1–G9) were exposed to either a *Microcystis* or a *Scenedesmus* diet. Bars indicate mean values, points indicate specific values for each population.

**FIGURE 4:**
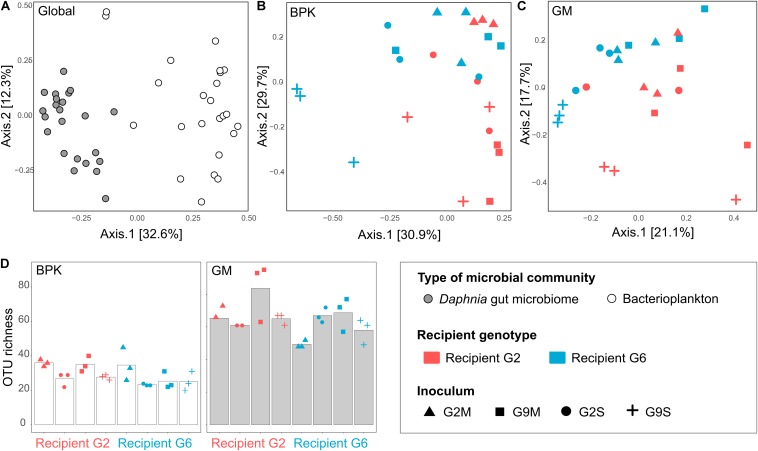
Contribution of recipient *Daphnia* genotype and microbial source to BPK and GM assembly in the transplant experiment (Experiment 2). **(A–C)** PCoA of microbial communities 2 weeks after the transplant, using Bray–Curtis dissimilarity indices, when considering all samples simultaneously **(A)**, BPK samples only **(B)**, and GM samples only **(C)**. **(D)** α-diversity (OTU richness) in the BPK and GM of recipient *Daphnia*. Bars indicate mean values, points indicate specific values for each population.

Our results show that *Daphnia* GM is not assembled randomly. There were strong differences between microbial communities in the *Daphnia* gut (GM) and in the surrounding water (BPK). In both experiments, the taxonomic composition of microbial communities (Figures 1, 2) differed between GM and BPK (Figures 3A, 4A and Tables 1a, 2a), mainly due to an overrepresentation of Actinobacteria (mostly Microbacteriaceae) and Flavobacteriia (*Flavobacterium* sp.) in BPK compared to GM (all *p* < 0.001 by Wald test; Supplementary Table S1). This suggests that these bacteria do not preferentially colonize the *Daphnia* gut environment. In Experiment 1, in which the cultures were regularly seeded with a diverse environmental sample of BPK, OTU richness was lower in GM than in BPK (*p* < 0.0001, GLM; Figure 3D and Supplementary Table S2a), suggesting a selection of particular BPK strains in the GM. β-proteobacteria (mostly Comamonadaceae) were over-represented in GM in Experiment 1 (*p* < 0.05 by Wald test; Supplementary Table S1a). The Comamonadaceae family is a major constituent of the microbiome and induces positive fitness effects in *Daphnia* (Eckert and Pernthaler, 2014; Peerakietkhajorn et al., 2015; Macke et al., 2017a; Callens et al., 2018; Mushegian et al., 2019). In Experiment 2, in which *Daphnia* were transferred into sterile water following the transplant so that BPK could only come from the leakage of *Daphnia* gut-associated microbes, OTU richness was higher in GM than in BPK (*p* < 0.0001, GLM; Figure 4D and Supplementary Table S3). This indicates that not all bacteria growing in *Daphnia* GM can thrive in an open environment. Important to note is that the GM is not the only potential host-derived source of microbiota. *Daphnia* contain microbiota on other structures as well, such as the carapace, the filter apparatus, etc. (Qi et al., 2009; Eckert and Pernthaler, 2014; Freese and Schink, 2011; Callens et al., 2016), which may explain the difference between GM and BPK. Taken together, our results suggest that under natural conditions with a diverse environmental microbial community, the gut microbiota will be a subset of the taxa occurring in the environment, while the taxa that leak from the GMs into the environment show differential capacities to contribute to the environmental microbial communities. Similar results were observed when considering the Shannon diversity index (Supplementary Tables S4, S5). Figures 3D, 4D suggest that α-diversity values were similar in both experiments for the GM, but not for the BPK, which was more diverse in the non-sterile medium of Experiment 1 than in the sterile medium of Experiment 2.

**TABLE 1:** Effects of community type, diet, and genotype on the taxonomic composition of microbial communities (Experiment 1).

Variable	*Df*	Sum of squares	Mean squares	F model	*R*^2^	Pr (>*F*)	Corrected *p*-value
**(a) Global PERMANOVA (BPK + GM)**							
Community type	1	7.88	7.88	39.41	0.21	0.001	0.0014
Diet	1	1.26	1.26	6.32	0.03	0.001	0.0014
Genotype	8	5.71	0.71	3.57	0.15	0.001	0.0014
Community × diet	1	0.95	0.95	4.77	0.02	0.001	0.0014
Community × genotype	8	4.99	0.62	3.12	0.13	0.001	0.0014
Diet × genotype	8	2.04	0.25	1.27	0.05	0.058	0.068
Community × diet × genotype	8	1.71	0.21	1.07	0.04	0.285	0.285
Residuals	68	13.59	0.20		0.36		
Total	103	38.14			1.00		
**(b) PERMANOVA in BPK**							
Diet	1	1.70	1.70	7.97	0.11	0.001	
Genotype	8	3.33	0.42	1.95	0.22	0.001	
Diet × genotype	8	2.80	0.35	1.64	0.19	0.001	
Residuals	34	7.26	0.21		0.48		
Total	51	15.10			1.00		
**(c) PERMANOVA in GM**							
Diet	1	0.53	0.53	2.85	0.04	0.029	
Genotype	8	7.36	0.92	4.94	0.49	0.001	
Diet × genotype	8	0.94	0.12	0.63	0.06	0.962	
Residuals	34	6.33	0.19		0.41		
Total	51	15.16			1.00		

*PERMANOVA were performed on Bray–Curtis dissimilarity indices for BPK and GM simultaneously **(a)**, BPK only **(b)**, and GM only **(c)**. *p*-values in the global analysis (a) were adjusted with the FDR method. Significant results (*p* < 0.05) are indicated in bold. Overall, the diet effect was stronger on BPK, while GM composition was mostly affected by host genotype, suggesting that genotype-dependent sorting processes are more important in GM than in BPK. However, it should be noted that the genotype effect is mainly driven by the strong differentiation of the G3 and G9 genotypes. When these two highly divergent genotypes are not included in the analysis, the diet remains the only significant factor affecting GM composition (*p* = 0.04).*

**TABLE 2:** Effects of community type, microbiome inoculum, and recipient genotype on the taxonomic composition of microbial communities (Experiment 2).

Variable	*Df*	Sum of squares	Mean squares	*F* model	*R*^2^	Pr (>*F*)	Corrected *p*-value
**(a) General PERMANOVA (BPK + GM)**							
Community type	1	4.05	4.05	30.02	0.28	0.001	0.002
Recipient genotype	1	0.79	0.79	5.87	0.05	0.002	0.003
Inoculum	3	2.05	0.68	5.05	0.14	0.001	0.002
Community × recipient genotype	1	0.64	0.64	4.71	0.04	0.001	0.002
Community × inoculum	3	1.20	0.40	2.95	0.08	0.002	0.003
Recipient genotype × inoculum	3	0.93	0.31	2.30	0.06	0.005	0.006
Community × recipient × inoculum	3	0.73	0.24	1.81	0.05	0.027	0.027
Residuals	31	4.19	0.16		0.29		
Total	46	14.57			1.00		
**(b) PERMANOVA in BPK**							
Recipient genotype	1	0.81	0.81	7.65	0.15	0.001	
Inoculum	3	1.77	0.59	5.59	0.34	0.001	
Recipient genotype × inoculum	3	0.95	0.32	3.00	0.18	0.003	
Residuals		1.69	0.11		0.32		
Total		5.22			1.00		
**(c) PERMANOVA in GM**							
Recipient genotype	1	0.62	0.62	3.72	0.12	0.001	
Inoculum	3	1.47	0.49	2.95	0.28	0.001	
Recipient genotype × inoculum	3	0.72	0.24	1.43	0.13	0.044	
Residuals	15	2.50	0.17		0.47		
Total	22	5.30			1.00		

*PERMANOVA were performed on Bray–Curtis dissimilarity indices for BPK and GM simultaneously **(a)**, BPK only **(b)**, and GM only **(c)**. *p*-values in the general analysis (a) were adjusted with the FDR method. Significant results (*p* < 0.05) are indicated in bold.*

Another conclusion of our study is that the host genotype influences the composition of both the GM and BPK community. In Experiment 1, after ±58 generations of exposure of *Daphnia* to a shared environmental microbial pool, both GM and BPK compositions were influenced by *Daphnia* genotype. In Experiment 2, 2 weeks after a GM transplant, the two recipient genotypes differed in terms of both GM and BPK compositions. Host-mediated structuring of the external microbial environment has already been reported in *Drosophila* (Wong et al., 2015) and in *Daphnia* (Degans et al., 2002). Our study reveals that this impact can be genotype-dependent. In this way, our results indicate that the composition of the environmental microbial community is affected by the evolution of the host species (see also terhorst et al., 2014), providing a potentially important avenue for future research in the field of eco-evolutionary dynamics (Hendry, 2017; De Meester et al., 2019). *Daphnia*-mediated modification of the BPK might further influence the success of species in the freshwater community and induce ecosystem-level effects. In Experiment 1, the overall β-diversity of microbial communities was affected by host diet and genotype as well as by the interaction of these two factors (Table 1a). However, diet and genotype effects on BPK were different from those on GM (i.e., significant interactions with the community type; Table 1a). A Mantel test (*R* = −0.025, *p* = 0.68) and Procrustes analysis (Pr: m^2^ = 0.96, *r* = 0.19, *p* = 0.32; Figures 3B,C) revealed no association between community composition in GM and BPK. Focusing on BPK separately, both diet and host genotype had a substantial effect on the taxonomic composition, and as these two factors interacted (Figure 3B and Table 1b), this suggests that the effect of diet varied among genotypes. Figure 3B indicates that the genotype effect was stronger with the *Microcystis* than with the *Scenedesmus* diet. Overall, diet had the strongest impact on the proportion of Flavobacteriia and Sphingobacteriia, which were overrepresented in *Daphnia* on the *Microcystis* diet compared to those on the *Scenedesmus* diet (both *p* < 0.01, by Wald test; Supplementary Table S6). In GM, the taxonomic composition was mainly affected by the genotype, while the diet had a smaller effect (Figure 3C and Table 1c). Flavobacteriia, Cytophagia, Actinobacteria, and Bacilli were all more strongly associated with the *Microcystis* diet, whereas α- and γ-proteobacteria were more strongly associated with the *Scenedesmus* diet (all *p* < 0.05 by Wald test; Supplementary Table S6). The effects of genotype and diet on OTU richness were dependent on the community type (*p* < 0.0002 for all interactions involving the community type, GLM; Figure 3D and Supplementary Tables S2a, S7). In BPK, OTU richness differed among genotypes (*p* < 0.0001, GLM; Figure 3D and Supplementary Table S3b), and the diet had a genotype-dependent impact (Diet: *p* = 0.0042, diet × genotype: *p* < 0.0001, GLM; Figure 3D and Supplementary Table S2b). In GM, OTU richness was affected by genotype (*p* < 0.0001, GLM; Figure 3D and Supplementary Table S2c), diet (*p* = 0.048, GLM; Figure 3D and Supplementary Table S2c) and their interaction (*p* < 0.0001, GLM; Figure 3D and Supplementary Table S2c). The Shannon diversity index was influenced by host genotype in both BPK and GM (*p* = 0.005, GLM; Supplementary Tables S4, S8), but it was not influenced by the diet (*p* = 0.45, GLM; Supplementary Tables S4, S8).

**FIGURE 5:**
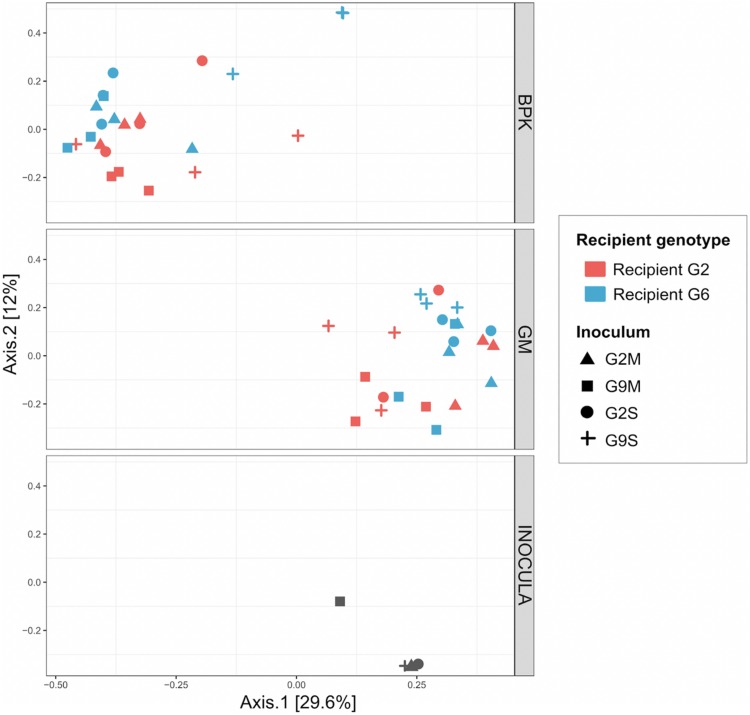
Principal coordinate analysis of microbial communities two weeks after the transplant, using Bray–Curtis dissimilarity indices (Experiment 2). Here, the microbiome inocula used to perform the transplant experiment were included in the analysis. BPK samples, GM samples and microbiome inocula were analyzed together, but are represented on different panels to facilitate interpretation.

The external microbial source interacts with the *Daphnia* genotype to shape GM and BPK. In addition to host-dependent processes, our results revealed that GM and BPK are affected by external factors including the diet (Experiment 1) and the external microbial source (Experiment 2). Both factors interacted with host genotype to shape the microbial communities. Overall, the diet effect was stronger in BPK, while GM composition was mostly affected by host genotype, suggesting that genotype-dependent sorting processes are more important in GM than in BPK. Exposure to *Microcystis* tended to decrease α-diversity in both types of communities and was associated with particular taxonomic groups, mainly Flavobacteria. Flavobacteria contribute to the lysis and degradation of *Microcystis* cells during cyanobacterial blooms (Maruyama et al., 2003; Kolmonen et al., 2004; Eiler and Bertilsson, 2007; Manage and Premetilake, 2011) and were found to be associated with *Microcystis* tolerance in *Daphnia* (Macke et al., 2017a). Interestingly, our results suggest that the structuring effect of host genotype on BPK is stronger with the *Microcystis* diet. Overall, the taxonomic composition of microbial communities was affected by both recipient genotype and microbiome inoculum (Figures 2, 4B,C and Table 2a). Differences among recipient genotypes and among microbiome inocula, however, varied depending on the community type (*p* < 0.03 for all interactions involving the community type, PERMANOVA; Table 2a). In both BPK and GM, both recipient genotype and microbiome inoculum affected the community structure, and both factors interacted (Figures 4B,C,5 and Tables 2b,c). The results of a Mantel test (*R* = 0.38, *p* = 0.002) and a Procrustes analysis (m^2^ = 0.59, *r* = 0.64, *p* = 0.001; Figures 4B,C) showed that variation in community composition among experimental treatments covaried between BPK and GM. OTU richness differed between the two recipient genotypes, clone G2 having a higher OTU richness than clone G6 for both BPK and GM (*p* = 0.003, GLM; Figure 4D and Supplementary Table S3). OTU richness was also affected by the microbiome inoculum (*p* = 0.009, GLM; Figure 4D and Supplementary Table S3), and this effect differed among community types (*p* = 0.002; Supplementary Table S3). The Shannon diversity index, however, was not affected by these factors (all *p* > 0.1, GLM; Supplementary Table S5).

*Daphnia* feeding behavior can play a role in structuring *Daphnia*-associated microbial communities (Degans et al., 2002; Mushegian et al., 2019). More specifically, by mediating the intake of microbes from the environment, *Daphnia* grazing activity might directly affect the relative abundance of certain bacterial strains both within the gut and in the BPK. Differential grazing might also influence microbial community composition indirectly, through its effect on phytoplankton. Algae produce exopolysaccharides and metabolites that are used by BPK and may influence their relative abundance (Taylor et al., 2014). By feeding on algae, *Daphnia* might affect the resources available for bacteria both in the BPK and in the GM and thus affect the community structure. Second, by shaping the intestinal environment, host metabolism and immunity, might contribute to drive GM assembly and favor the growth of specific microbes within the gut (Macke et al., 2017b). These effects may further extend to influence the external environment of *Daphnia*, for instance through the shedding of immune effectors (Otti et al., 2014), thus affecting the BPK community structure. Third, GM communities might influence environmental microbial communities by leakage of dominant taxa. If hosts are common in the environment, such leakage might be an important force in structuring free-living microbial communities. In Experiment 2, we found a positive correlation between variation in GM composition and variation in BPK composition. This either reflects a shared structuring effect of *Daphnia* on both GM and BPK, or a direct interaction between GM and BPK. This association was not present in Experiment 1, likely because it was masked in the presence of a more diverse environmental microbial community. Given the low congruence of GM and BPK community structure at the OTU level, it is, however, unlikely that GM contributed strongly to the structuring of BPK via the leakage of microbes, although some seeding cannot be excluded. Another explanation that does not discount the role of leakage of bacterial strains from the gut into the environment could be the different growth rate of bacteria in the gut versus the water. If some microbes have a higher growth rate in the gut than in the environment, this could lead to differences between GM and BPK, suggesting that some microbes, which are rare in the environment but common in the gut, may only be maintained by leakage from the host. Alternatively, GM may have contributed to structuring BPK indirectly via the expression of particular metabolites. Microbe–microbe interactions are indeed central in structuring microbial communities, more specifically through the production of antimicrobial molecules (Tasiemski et al., 2015; Adair and Douglas, 2017).

We can conclude by stating that host-dependent processes interact with external factors to shape both the GM and wider bacterial environment, suggesting that these two communities influence each other. Interactions between host genotypes, their GMs and free-living microbial communities might have important ecosystem level effects. This structuring effect is an essential prerequisite for the important role of the microbiome in eco-evolutionary processes and generates adaptive flexibility. An important avenue for future studies will be to determine how the microbiome can affect interactions among host species, e.g., by measuring the impact of microbial dispersal on the GMs, as well as on their fitness and competitive strength.

## Data Availability Statement

The datasets generated for this study can be found in the NCBI, under accession numbers PRJNA498431 and PRJNA498417.

## Author Contributions

EM, MC, ED, and LD conceived the ideas and designed the experiment approach. EM, MC, IV, and ED performed the experiments, did the sequencing and collected the data. EM, MC, and FM analyzed the data, with input from ED and LD. EM and ED then led the writing of the manuscript. All authors contributed to the revised versions.

## Conflict of Interest

The authors declare that the research was conducted in the absence of any commercial or financial relationships that could be construed as a potential conflict of interest.
