# Supplementary material for: Diet and Genotype of an Aquatic Invertebrate Affect the Composition of Free-Living Microbial Communities
Source: Front Microbiol. 2020 Mar 17;11:380. doi: 10.3389/fmicb.2020.00380 (PMC7090131; doi:10.3389/fmicb.2020.00380)
Supplement: Supplementary file 1 [file Data_Sheet_1.docx]

**Table S1. Bacterial classes differing between BPK and GM in Experiment 1 (a) and Experiment 2 (b).** A differential abundance analysis was performed with the Bioconductor package DESeq2. Results indicate the log2-fold change in GM compared to BPK. IfcSE is the standard error for the log2 fold-change estimate. Significant results (p<0.05) are indicated in bold. In Experiment 1, the analysis was performed separately for each diet, and genotypes G3 and G9 (which had a very different profile) were discarded from the analysis.

| **a) Experiment 1** | | | | | | | |
| --- | --- | --- | --- | --- | --- | --- | --- |
| Diet | Class | Base mean | log2  Fold-change | lfcSE | Wald stat. | pvalue | padj |
| M | Betaproteobacteria | 12831.38 | 0.59 | 0.23 | 2.55 | 1.07E-02 | **1.92e-02** |
|  | Flavobacteriia | 4010.27 | -4.38 | 0.60 | -7.31 | 2.71E-13 | **1.22e-12** |
|  | Actinobacteria | 4141.11 | -2.08 | 0.55 | -3.76 | 1.68E-04 | **5.03e-04** |
|  | Planctomycetia | 524.55 | 1.35 | 0.62 | 2.17 | 2.97E-02 | **4.46e-02** |
|  | Cytophagia | 390.02 | 1.74 | 0.63 | 2.77 | 5.64E-03 | **1.27e-02** |
|  | Bacilli | 254.16 | 9.41 | 0.95 | 9.94 | 2.74E-23 | **2.46e-22** |
| S | Betaproteobacteria | 22874.49 | 1.44 | 0.38 | 3.81 | 1.40E-04 | **1.64e-04** |
|  | Flavobacteriia | 522.68 | -2.98 | 0.43 | -6.88 | 5.87E-12 | **4.11e-11** |
|  | Actinobacteria | 4651.27 | -3.08 | 0.48 | -6.37 | 1.85E-10 | **6.47e-10** |
|  | Sphingobacteriia | 1471.52 | 2.72 | 0.60 | 4.51 | 6.53E-06 | **9.14e-06** |
|  | Planctomycetia | 702.57 | 2.03 | 0.42 | 4.81 | 1.54E-06 | **2.69e-06** |
|  | Gammaproteobacteria | 1260.94 | 2.22 | 0.45 | 4.93 | 8.06E-07 | **1.88e-06** |
|  | Alphaproteobacteria | 802.02 | 1.38 | 0.48 | 2.90 | 3.70E-03 | **3.70e-03** |
| **b) Experiment 2** | | | | | | | |
| Class | | Base mean | log2  Fold-change | lfcSE | Wald stat. | pvalue | padj |
| Betaproteobacteria | | 20008.74 | -1.37 | 0.30 | -4.62 | 3.89E-06 | **5.83e-06** |
| Gammaproteobacteria | | 998.50 | 1.40 | 0.25 | 5.61 | 1.99E-08 | **4.48e-08** |
| Sphingobacteriia | | 415.01 | -1.46 | 0.54 | -2.69 | 7.25E-03 | **9.32e-03** |
| Actinobacteria | | 2469.88 | -3.44 | 0.66 | -5.24 | 1.56E-07 | **2.82e-07** |
| Flavobacteriia | | 5465.56 | -8.43 | 0.63 | -13.36 | 1.08E-40 | **9.75e-40** |
| Bacilli | | 359.13 | 6.93 | 0.74 | 9.37 | 7.37E-21 | **2.21e-20** |
| Planctomycetia | | 133.63 | 7.53 | 0.66 | 11.36 | 6.32E-30 | **2.84e-29** |

**Table S2. Effects of community type, diet and genotype on OTU richness (Experiment 1).** A GLM model (distribution = Poisson) was applied for BPK and GM simultaneously **(a)**, BPK only **(b)** and GM only **(c)**. In the global analysis (a) p-values were adjusted for multiple comparisons through the control of the false discovery rate (FDR). Significant results (p<0.05) are indicated in bold.

| **a) Global GLM (BPK + GM)** | | | | | | |
| --- | --- | --- | --- | --- | --- | --- |
| Variable | Df | Deviance | Residual Df | Residual deviance | Pr(>Chi) | Corrected p-value |
| NULL |  |  | 103 | 1667.2 |  |  |
| Community type | 1 | 697.83 | 102 | 969.37 | 8.88e-154 | **6.22e-153** |
| Diet | 1 | 1.30 | 101 | 968.07 | 0.25 | 0.25 |
| Genotype | 8 | 154.40 | 93 | 813.67 | 2.36e-29 | **8.27e-29** |
| Community*Diet | 1 | 14.6 | 92 | 799.07 | 0.00013 | **0.00015** |
| Community*Genotype | 8 | 133.01 | 84 | 666.06 | 6.71e-25 | **1.57e-24** |
| Diet*Genotype | 8 | 119.37 | 76 | 546.69 | 4.48e-22 | **7.84e-22** |
| Community*Diet*Genotype | 8 | 44.77 | 68 | 501.92 | 4.066e-07 | **5.69e-07** |
| **b) GLM in BPK** | | | | | | |
| Variable | Df | Deviance | Residual Df | Residual deviance | Pr(>Chi) | |
| NULL |  |  | 51 | 502.27 |  | |
| Diet | 1 | 8.20 | 50 | 494.07 | **0.0042** | |
| Genotype | 8 | 68.43 | 42 | 425.64 | **1.009e-11** | |
| Diet*Genotype | 8 | 97.96 | 34 | 327.68 | **1.12e-17** | |
| **c) GLM in GM** | | | | | | |
| Variable | Df | Deviance | Residual Df | Residual deviance | Pr(>Chi) | |
| NULL |  |  | 51 | 467.10 |  | |
| Diet | 1 | 3.92 | 50 | 463.19 | **0.048** | |
| Genotype | 8 | 222.77 | 42 | 240.42 | **1.003e-43** | |
| Diet*Genotype | 8 | 66.18 | 34 | 174.24 | **2.819e-11** | |

**Table S3. Effects of community type, microbiome inoculum and recipient genotype on OTU richness (Experiment 2).** A GLM model (distribution = Poisson) was applied. P-values were adjusted for multiple comparisons through the control of the false discovery rate. Significant results (p<0.05) are indicated in bold.

| Variable | Df | Deviance | Residual Df | Residual deviance | Pr(>Chi) | Corrected p-value |
| --- | --- | --- | --- | --- | --- | --- |
| NULL |  |  | 46 | 405.88 |  |  |
| Community type | 1 | 327.16 | 45 | 78.72 | 3.99e-73 | **2.80e-72** |
| Recipient genotype | 1 | 10.39 | 44 | 68.33 | 0.0013 | **0.003** |
| Inoculum | 3 | 12.85 | 41 | 55.48 | 0.005 | **0.009** |
| Community*Rec. gen. | 1 | 0.03 | 40 | 55.45 | 0.86 | 0.86 |
| Community*Inoculum | 3 | 17.84 | 37 | 37.61 | 0.0005 | **0.002** |
| Rec. gen.*Inoculum | 3 | 4.72 | 34 | 32.89 | 0.19 | 0.27 |
| Com.*Rec. gen.*Inoc. | 3 | 3.71 | 31 | 29.18 | 0.29 | 0.34 |

**Table S4. Effects of community type, diet and genotype on Shannon diversity (Experiment 1).** A GLM model (distribution = Gaussian) was applied for BPK and GM simultaneously **(a)**, BPK only **(b)** and GM only **(c)**. In the global model (a) p-values were adjusted for multiple comparisons through the control of the false discovery rate (FDR). Significant results (p<0.05) are indicated in bold.

| **a) Global GLM (BPK + GM)** | | | | | | | |
| --- | --- | --- | --- | --- | --- | --- | --- |
| Variable | Df | Dev. | Residual Df | Residual dev. | F | Pr(>F) | Corrected p-value |
| NULL |  |  | 103 | 8779.4 |  |  |  |
| Community type | 1 | 2858.53 | 102 | 5920.3 | 77.01 | 8.6e-13 | **6.02e-12** |
| Diet | 1 | 21.56 | 101 | 5899.3 | 0.58 | 0.449 | 0.449 |
| Genotype | 8 | 1029.15 | 93 | 4870.2 | 3.47 | 0.002 | **0.005** |
| Community*Diet | 1 | 25.25 | 92 | 4844.9 | 0.68 | 0.412 | 0.449 |
| Community*Genotype | 8 | 584.31 | 84 | 4260.6 | 1.97 | 0.064 | 0.089 |
| Diet*Genotype | 8 | 1094.99 | 76 | 3165.6 | 3.69 | 0.001 | **0.004** |
| Community*Diet*Genotype | 8 | 641.41 | 68 | 2524.2 | 2.16 | 0.041 | 0.073 |
| **b) GLM in BPK** | | | | | | | |
| Variable | Df | Dev. | Residual Df | Residual dev. | F | Pr(>F) | |
| NULL |  |  | 51 | 4987.3 |  |  | |
| Diet | 1 | 32.33 | 50 | 4955.0 | 0.56 | 0.46 | |
| Genotype | 8 | 1294.51 | 42 | 3660.5 | 2.81 | **0.017** | |
| Diet*Genotype | 8 | 1701.88 | 34 | 1958.6 | 3.69 | **0.003** | |
| **c) GLM in GM** | | | | | | | |
| Variable | Df | Dev. | Residual Df | Residual dev. | F | Pr(>F) | |
| NULL |  |  | 51 | 933.54 |  |  | |
| Diet | 1 | 0.77 | 50 | 932.76 | 0.047 | 0.83 | |
| Genotype | 8 | 332.66 | 42 | 600.1 | 2.50 | **0.03** | |
| Diet*Genotype | 8 | 34.52 | 34 | 565.57 | 0.26 | 0.97 | |

**Table S5. Effects of community type, microbiome inoculum and recipient genotype on Shannon diversity (Experiment 2).** A GLM model (distribution = Gaussian) was applied. P-values were adjusted for multiple comparisons through the control of the false discovery rate. Significant results (p<0.05) are indicated in bold.

| Variable | Df | Dev. | Residual Df | Residual dev. | F | Pr(>F) | Corrected p-value |
| --- | --- | --- | --- | --- | --- | --- | --- |
| NULL |  |  | 46 | 951.98 |  |  |  |
| Community type | 1 | 303.30 | 45 | 648.68 | 27.93 | 9.48e-06 | **6.64e-05** |
| Recipient genotype | 1 | 0.21 | 44 | 648.47 | 0.02 | 0.89 | 0.89 |
| Inoculum | 3 | 41.89 | 41 | 606.58 | 1.29 | 0.30 | 0.38 |
| Community*Rec. gen. | 1 | 10.64 | 40 | 595.95 | 0.98 | 0.33 | 0.38 |
| Community*Inoculum | 3 | 59.00 | 37 | 536.95 | 1.81 | 0.17 | 0.29 |
| Rec. gen.*Inoculum | 3 | 97.43 | 34 | 439.51 | 2.99 | 0.046 | 0.11 |
| Com.*Rec. gen.*Inoc. | 3 | 102.91 | 31 | 336.60 | 3.16 | 0.038 | 0.11 |

**Table S6. Bacterial classes differing between the *Microcystis* and the *Scenedesmus* diets (Experiment 1).** A differential abundance analysis was performed with the Bioconductor package DESeq2, separately for each community type. Because genotypes G3 and G9 had a very different profile, they were discarded from the analysis. Results indicate the log2-fold change in the *Scenedesmus* diet compared to the *Microcystis* diet. Significant results (p<0.05) are indicated in bold.

| Community | Class | Base mean | log2  Fold-change | lfcSE | Wald stat. | pvalue | padj |
| --- | --- | --- | --- | --- | --- | --- | --- |
| BPK | Flavobacteriia | 5092.19 | -3.13 | 0.53 | -5.93 | 3.03e-09 | **2.12e-08** |
|  | Sphingobacteriia | 1131.83 | -1.65 | 0.52 | -3.18 | 1.48E-03 | **0.0052** |
| GM | Flavobacteriia | 226.42 | -1.78 | 0.53 | -3.37 | 0.001 | **0.002** |
|  | Actinobacteria | 1241.23 | -1.44 | 0.56 | -2.57 | 0.010 | **0.023** |
|  | Gammaproteobact. | 1063.83 | 1.13 | 0.47 | 2.39 | 0.017 | **0.025** |
|  | Cytophagia | 382.23 | -2.51 | 0.72 | -3.49 | 0.000 | **0.002** |
|  | Alphaproteobact. | 706.84 | 2.30 | 0.66 | 3.51 | 0.000 | **0.002** |
|  | Bacilli | 376.40 | -1.38 | 0.56 | -2.46 | 0.014 | **0.025** |

**Table S7. Pairwise comparisons of OTU richness among genotypes and diets in BPK and GM (Experiment 1).** Contrasts were computed on least-squares means, separately for each community type. Results are given on the log scale. P-values were adjusted with the FDR method. Letters (Group) indicate significant differences (p<0.05).

| Community | Diet | Genotype | Lsmean | SE | df | Asymp.LCL | Asymp.UCL | Group |
| --- | --- | --- | --- | --- | --- | --- | --- | --- |
| BPK | M | G6 | 4.19 | 0.07 | NA | 3.98 | 4.41 | a |
|  | M | G9 | 4.24 | 0.07 | NA | 4.03 | 4.45 | ab |
|  | M | G8 | 4.29 | 0.07 | NA | 4.08 | 4.49 | abc |
|  | M | G3 | 4.31 | 0.07 | NA | 4.11 | 4.51 | abc |
|  | M | G5 | 4.44 | 0.08 | NA | 4.21 | 4.67 | bcd |
|  | S | G1 | 4.48 | 0.06 | NA | 4.29 | 4.66 | cd |
|  | S | G2 | 4.48 | 0.06 | NA | 4.29 | 4.66 | cd |
|  | S | G6 | 4.53 | 0.06 | NA | 4.35 | 4.71 | de |
|  | M | G1 | 4.53 | 0.06 | NA | 4.35 | 4.71 | def |
|  | S | G9 | 4.56 | 0.06 | NA | 4.39 | 4.74 | def |
|  | S | G7 | 4.57 | 0.06 | NA | 4.40 | 4.75 | def |
|  | M | G4 | 4.58 | 0.06 | NA | 4.41 | 4.76 | def |
|  | S | G8 | 4.61 | 0.06 | NA | 4.43 | 4.78 | def |
|  | S | G3 | 4.69 | 0.06 | NA | 4.53 | 4.86 | efg |
|  | S | G5 | 4.71 | 0.07 | NA | 4.50 | 4.91 | efgh |
|  | S | G4 | 4.71 | 0.05 | NA | 4.55 | 4.87 | fg |
|  | M | G7 | 4.83 | 0.05 | NA | 4.68 | 4.99 | gh |
|  | M | G2 | 4.89 | 0.05 | NA | 4.74 | 5.04 | h |
| GUT | S | G3 | 2.73 | 0.15 | NA | 2.29 | 3.17 | a |
|  | M | G3 | 3.60 | 0.10 | NA | 3.32 | 3.89 | b |
|  | M | G9 | 3.66 | 0.09 | NA | 3.39 | 3.94 | bc |
|  | S | G9 | 3.70 | 0.09 | NA | 3.43 | 3.97 | bc |
|  | M | G4 | 3.75 | 0.09 | NA | 3.49 | 4.02 | bcd |
|  | S | G4 | 3.84 | 0.08 | NA | 3.58 | 4.09 | bcde |
|  | S | G7 | 3.88 | 0.08 | NA | 3.63 | 4.13 | bcde |
|  | M | G6 | 3.91 | 0.08 | NA | 3.66 | 4.15 | cde |
|  | M | G1 | 3.93 | 0.08 | NA | 3.69 | 4.17 | cde |
|  | S | G1 | 4.00 | 0.08 | NA | 3.76 | 4.23 | de |
|  | S | G5 | 4.01 | 0.08 | NA | 3.77 | 4.24 | def |
|  | S | G8 | 4.02 | 0.08 | NA | 3.79 | 4.25 | def |
|  | S | G6 | 4.03 | 0.08 | NA | 3.79 | 4.26 | ef |
|  | M | G8 | 4.03 | 0.08 | NA | 3.80 | 4.26 | ef |
|  | M | G7 | 4.04 | 0.08 | NA | 3.81 | 4.27 | ef |
|  | M | G5 | 4.08 | 0.07 | NA | 3.86 | 4.31 | ef |
|  | S | G2 | 4.24 | 0.07 | NA | 4.04 | 4.45 | f |
|  | M | G2 | 5.00 | 0.08 | NA | 4.76 | 5.25 | g |

**Table S8. Pairwise comparisons of the Shannon diversity index among genotypes and diets in BPK and GM (Experiment 1).** Contrasts were computed on least-squares means, separately for each community type. P-values were adjusted with the FDR method. Letters (Group) indicate significant differences (p<0.05).

| Community | Diet | Genotype | Lsmean | SE | df | Asymp.LCL | Asymp.UCL | Group |
| --- | --- | --- | --- | --- | --- | --- | --- | --- |
| BPK | M | G6 | 8.86 | 4.38 | NA | -4.25 | 21.96 | a |
|  | M | G9 | 9.46 | 4.38 | NA | -3.64 | 22.57 | ab |
|  | M | G1 | 9.59 | 4.38 | NA | -3.52 | 22.70 | ab |
|  | M | G3 | 10.14 | 4.38 | NA | -2.96 | 23.25 | ab |
|  | M | G5 | 11.03 | 5.37 | NA | -5.02 | 27.09 | abc |
|  | S | G1 | 11.09 | 4.38 | NA | -2.02 | 24.20 | abc |
|  | M | G4 | 11.18 | 4.38 | NA | -1.93 | 24.29 | abc |
|  | S | G8 | 11.99 | 4.38 | NA | -1.12 | 25.10 | abc |
|  | S | G6 | 12.68 | 4.38 | NA | -0.43 | 25.79 | abc |
|  | M | G8 | 13.58 | 4.38 | NA | 0.47 | 26.69 | abc |
|  | S | G7 | 14.68 | 4.38 | NA | 1.57 | 27.79 | abc |
|  | S | G2 | 15.91 | 4.38 | NA | 2.80 | 29.01 | abc |
|  | S | G4 | 16.75 | 4.38 | NA | 3.65 | 29.86 | abc |
|  | S | G9 | 17.92 | 4.38 | NA | 4.81 | 31.03 | abc |
|  | M | G7 | 27.20 | 4.38 | NA | 14.09 | 40.31 | cd |
|  | S | G3 | 27.24 | 4.38 | NA | 14.13 | 40.35 | cd |
|  | S | G5 | 27.87 | 5.37 | NA | 11.82 | 43.93 | bcd |
|  | M | G2 | 35.81 | 4.38 | NA | 22.70 | 48.91 | d |
| GM | - | G3 | 1.93 | 1.67 | NA | -2.68 | 6.55 | a |
|  | - | G9 | 3.00 | 1.67 | NA | -1.62 | 7.61 | ab |
|  | - | G1 | 4.42 | 1.67 | NA | -0.20 | 9.04 | ab |
|  | - | G4 | 5.09 | 1.67 | NA | 0.48 | 9.71 | ab |
|  | - | G7 | 5.31 | 1.67 | NA | 0.70 | 9.93 | ab |
|  | - | G5 | 6.24 | 1.67 | NA | 1.63 | 10.86 | abc |
|  | - | G8 | 7.08 | 1.67 | NA | 2.46 | 11.70 | abc |
|  | - | G6 | 8.39 | 1.67 | NA | 3.77 | 13.00 | bc |
|  | - | G2 | 12.92 | 2.35 | NA | 6.40 | 19.45 | c |
